# Trialling a microbiome-targeted dietary intervention in children with ADHD—the rationale and a non-randomised feasibility study

**DOI:** 10.1186/s40814-022-01058-4

**Published:** 2022-05-23

**Authors:** Kate Lawrence, Kyriaki Myrissa, Miguel Toribio-Mateas, Lori Minini, Alice M. Gregory

**Affiliations:** 1 Department of Psychology & Pedagogic Science, Faculty of Sport, Allied Health and Performance Science, St Mary’s University, Twickenham, London, UK; 2 Department of Health Sciences, Faculty of Sport, Allied Health and Performance Science, St Mary’s University, Twickenham, London, UK; 3 School of Health and Education, Middlesex University, London, UK; 4 School of Applied Science, London South Bank University, London, UK; 5 Department of Psychology, Goldsmiths, University of London, London, UK

**Keywords:** Actigraphy, Attention-deficit hyperactivity disorder, ADHD, Children, Diet, Feasibility study, Kefir, Microbiome, Microbiota, Sleep

## Abstract

**Background:**

Dietary interventions have been previously explored in children with ADHD. Elimination diets and supplementation can produce beneficial behaviour changes, but little is known about the mechanisms mediating change. We propose that these interventions may work, in part, by causing changes in the gut microbiota. A microbiome-targeted dietary intervention was developed, and its feasibility assessed.

**Methods:**

A non-randomised feasibility study was conducted on nine non-medicated children with ADHD, aged 8–13 years (mean 10.39 years), using a prospective one-group pre-test/post-test design. Participants were recruited from ADHD support groups in London and took part in the 6-week microbiome-targeted dietary intervention, which was specifically designed to impact the composition of gut bacteria. Children were assessed pre- and post-intervention on measures of ADHD symptomatology, cognition, sleep, gut function and stool-sample microbiome analysis. The primary aim was to assess the study completion rate, with secondary aims assessing adherence, adverse events (aiming for no severe and minimal), acceptability and suitability of outcome measures.

**Results:**

Recruitment proved to be challenging and despite targeting 230 participants directly through support groups, and many more through social media, nine families (of the planned 10) signed up for the trial. The completion rate for the study was excellent at 100%. Exploration of secondary aims revealed that (1) adherence to each aspect of the dietary protocol was very good; (2) two mild adverse events were reported; (3) parents rated the treatment as having good acceptability; (4) data collection and outcome measures were broadly feasible for use in an RCT with a few suggestions recommended; (5) descriptive data for outcome measures is presented and suggests that further exploration of gut microbiota, ADHD symptoms and sleep would be helpful in future research.

**Conclusions:**

This study provides preliminary evidence for the feasibility of a microbiome-targeted dietary intervention in children with ADHD. Recruitment was challenging, but the diet itself was well-tolerated and adherence was very good. Families wishing to trial this diet may find it an acceptable intervention. However, recruitment, even for this small pilot study, was challenging. Because of the difficulty experienced recruiting participants, future randomised controlled trials may wish to adopt a simpler dietary approach which requires less parental time and engagement, in order to recruit the number of participants required to make meaningful statistical interpretations of efficacy.

**Trial registration:**

ClinicalTrials.gov Identifier: NCT03737877. Registered 13 November 2018—retrospectively registered, within 2 days of the first participant being recruited.

**Supplementary Information:**

The online version contains supplementary material available at 10.1186/s40814-022-01058-4.

## Key messages regarding feasibility

1. What uncertainties existed regarding the feasibility?

Would we be able to recruit families to be involved in the study? Would participants be able to complete the 6-week dietary intervention? What would adherence rates be to each aspect of the dietary protocol? Would there be any adverse events? Would the treatment be acceptable to parents? Would data collection and outcome measures be appropriate for use in an RCT?

2) What are the key feasibility findings?

The diet itself was a feasible intervention for those who took part. The completion rate was 100%, with good levels of adherence and acceptability. Two mild adverse events were reported. The intervention had high levels of acceptability, adherence and completion. However, recruitment was very challenging meaning running an adequately powered RCT using this exact protocol is probably unfeasible.

3) What are the implications of the feasibility findings for the design of the main study?

Outcome measures were evaluated for their use in a larger trial and some recommendations suggested. Recruitment was challenging and it is suggested that a future larger-scale study may benefit from using a dietary intervention that requires less parental time and input, in order to recruit an adequate number of participants.

## Background

Attention-deficit/hyperactivity disorder (ADHD) is a neurodevelopmental disorder, defined by its behavioural symptoms of impulsivity, hyperactivity and inattention [1]. Prevalence rates are estimated to be rising with global, community prevalence in children estimated to be around 5% [2]. The main therapeutic options for children with ADHD are behavioural therapy, parent training and medication. According to some estimates, around two-thirds of children diagnosed with ADHD in the USA are on medication for their condition [3, 4], with prescriptions of Ritalin (Methylphenidate) increasing considerably over recent decades [5]. However, Methylphenidate does not always result in substantial symptom reduction and does not appear to reduce school drop-out rates or unemployment [6]. Furthermore, a number of debilitating side effects (such as anxiety, stomach ache, nausea, headaches and sleep problems) are experienced by a significant proportion of children taking Methylphenidate [7]. These, coupled with the long-term effects of stimulants on growth [8], have led parents to seek alternative treatments for their children that may have fewer side effects [9].

Parents keen to investigate non-pharmaceutical treatments are often drawn to considering dietary interventions. The main dietary interventions that have been explored in ADHD can be grouped into three broad categories: exclusion of food additives; essential fatty acid supplementation; and elimination diets. Stimulated by the early theoretical ideas of Feingold [10], numerous, predominantly small-scale, studies have explored the efficacy of excluding food additives, artificial colourings and flavourings, from the diet of children with ADHD [11, 12]. Meta-analyses report a significant, though modest, benefit of the exclusion of food additives on symptoms of ADHD [13, 14] although the results do not always remain significant when corrected for publication bias [13]. There has been widespread interest regarding the potential benefits of omega-3 polyunsaturated fatty acids (PUFAs) supplementation for individuals diagnosed with ADHD. Recent reviews suggest that approximately 50% of studies demonstrate a beneficial effect of PUFAs on ADHD symptoms with evidence overall remaining inconclusive [15, 16]. The Few Foods Diet is an elimination diet which excludes many commonly eaten foods, including those most likely to provoke sensitivities (such as gluten, dairy and citrus fruits). The ‘Few Foods Diet’ (sometimes referred to as an oligoantigenic diet) includes a highly restricted number of permitted foods (for example a diet that focuses exclusively on lamb, chicken, potatoes, rice, banana, apple and brassica) [17]. This diet can be highly effective at reducing symptoms in children with ADHD [17–20], evidenced by considerably larger effect sizes than alternative dietary treatments [16]. However, little is understood about the mechanisms underlying its success and its highly restrictive nature means it is relatively difficult to implement and adhere to.

Knowledge of the mechanisms by which these different dietary interventions may operate has not been fully established. We suggest that one means by which they may exert a behavioural change in children with ADHD is by manipulating the diversity and species of microorganisms residing within the gut. Ample evidence from both human and animal studies describes the impact of dietary interventions on the idiosyncrasies of the gut microbiota, the tens of trillions of microorganisms [21, 22] that inhabit our gastrointestinal system [23, 24]. Every unique community of microorganisms interacts with their human host through immune, neuroendocrine and neural pathways [25].

Research has shown that gut microbiota can have a profound impact on health [26–28], including mental health [29–32], potentially via its role in the gut-brain axis. The microbiome and the gut-brain axis have been implicated to play a role in many of the symptoms and characteristics which are commonly observed in individuals with ADHD, including anomalies with cognition [33–36], emotion [37, 38], anxiety [39] and sleep [40]. In addition, a number of risk factors associated with an increased prevalence in ADHD, including being born by cesarean [41], a lack of breastfeeding [42, 43] and maternal stress [44], are known to have a negative impact on the establishment of a healthy microbiome [45, 46]. The health of the microbiome has not been fully explored in children with ADHD, but there is some preliminary evidence to suggest that there may be some differences in the species of bacteria present in comparison to individuals without ADHD [47, 48], although the results are variable and the picture still rather unclear [49]. There are numerous plausible mechanisms for the way in which gut bacteria may influence the behavioural symptoms of children with ADHD. Microbiota have been reported to influence inflammation, the production of hormones (including the stress hormone, cortisol), neurotransmitters (such as serotonin) together with the development and function of brain structures such as the amygdala, prefrontal cortex and hippocampus, all of which could plausibly play a role in the development of ADHD [50–54].

Research has shown that gut bacteria can be manipulated in a matter of days by alterations to diet [55–57]. Therefore, the dietary interventions previously researched in ADHD may have exerted their effect, in part, by producing alterations in the microbiota. Looking specifically at the type of dietary interventions that have been trialled in children with ADHD, the consumption of artificial food additives has been linked to various shifts in microbiome communities [58, 59] with increasing evidence to suggest that artificial sweeteners and food additives may be linked to dysbiosis [59, 60]. Along with the role Omega 3 PUFAs have a role in decreasing inflammation [61] they can also be considered to be a type of prebiotic [62] and may exert a positive influence on the composition of the gut microbiota and consequently the gut-brain axis [62–64]. Finally, adopting a Few Foods diet would likely have a marked impact on microbiota composition; however, this is yet to be explored. The consumption of ultra-processed foods and high sugar diets have been linked to the proliferation of microbes that promote inflammatory disease [65, 66] and thus the removal of these foods may enhance the gut microbiota.

Whilst the dietary interventions explored in ADHD have the *potential* to improve the composition of the microbiota, to the best of our knowledge, none explicitly state that they were *designed* with this goal in mind. Diet can have a profound influence on the composition of gut bacteria and numerous studies attest to the potential benefit of specific microbiota targeted dietary interventions. Diets containing a rich diversity of plant fibre and polyphenols, provide fuel for the commensal bacteria in the gut and may also increase microbial diversity [67, 68] with the greater number of plant varieties consumed associated with greater microbial diversity [69]. Time-restricted eating can also enhance the diversity of the gut microbiome [70–72] and consuming fermented food, rich in probiotics, such as kefir, can produce an increase in beneficial gut bacteria such as *Lactobacillus* [73]. Nutritional education focusing on improving gut microbiota composition has been found to be associated with increased microbial diversity and improvements in physical and mental health in adult women [74]. Such an approach has not been trailed in ADHD but preliminary research trialling probiotics in this population suggests a potential benefit of microbiome targeted interventions [75, 76].

In this study, we propose a dietary intervention developed specifically for the purpose of targeting the gut microbiota and improving the microbial balance. From a behaviour change perspective, individuals typically find it easier to introduce new behaviours into their daily lives than they do to remove existing behaviours: habits become rather automatic, meaning breaking existing habits is more effortful and difficult than establishing new habits [77, 78]. This may be especially true for children with ADHD for whom difficulties with impulse control and behavioural inhibition are core symptoms of the disorder [79]. The dietary approach devised for the current study therefore centres largely on the introduction of positive changes in dietary behaviours, rather than the direct removal of existing behaviours.

The aim of this study was to assess the feasibility of the dietary intervention in order to support the development of a future randomised controlled trial (RCT). The study is reported in adherence to the CONSORT extension to pilot and feasibility studies [80, 81], and as such, there was no formal hypothesis testing using inferential statistics on the outcome measures [82]. The primary aim of the current study was to assess the feasibility of the dietary intervention in terms of the proportion of those who completed the dietary intervention. There were a number of secondary aims: (1) to assess percentage adherence to each aspect of the dietary protocol, (2) to record any adverse events, (3) to assess parent-rated acceptability of the treatment, (4) to evaluate data collection and outcome measures to assess their feasibility for use in an RCT, and (5) to explore the change in outcome measures for preliminary evidence of potential effects.

## Materials and methods

### Study design

This was a 6-week pilot feasibility study to assess the safety, tolerability, and compliance of a dietary intervention for children with ADHD. The feasibility study used a single-arm, pre-test/post-test design. This design was well suited to our study, which was investigating a new and innovative dietary intervention for which little information existed on the feasibility of the programme and the ability to carry out a large-scale trial. This trial was registered with ClinicalTrials.gov (Identifier: NCT03737877). Primary and secondary outcomes were measured through information collected throughout and at completion (week six) of the dietary intervention. Ethical approval for the study was granted by St Mary’s University Ethics Committee (SMEC_2017-18_132) and parents and children were provided with full written information about the study before written consent and assent were obtained. Participants completed baseline assessments at Time 1, prior to commencing the dietary intervention, and at Time 2, during week 6 of the diet.

### Participants

#### Inclusion and exclusion criteria

Children were eligible to take part in the study if they had received a diagnosis of ADHD by a specialist qualified healthcare professional and were aged between 8 years and 13 years at the onset of study. The lower limit was set at 8 years, as this is the youngest age at which self-report data can be gathered using the Conners Comprehensive Behavior Rating Scales [83] to measure ADHD symptomatology. An upper limit was set at 13 years as it was thought that parents would have less control of their child’s diet above this age which may impact adherence. Parental commitment to attending group sessions was also required. Males and females, children with co-occurring diagnoses and those with food allergies or sensitivities could take part in the study. Comorbidity is an epidemiological reality for many children with ADHD [84], and therefore, including such children allows us to represent the reality of an ADHD diagnosis. We were also primarily concerned about assessing the feasibility of following the diet rather than outcome measures.

Participants were not eligible to take part if they (1) were currently taking ADHD medication (such as Methylphenidate); (2) were currently undergoing a course of behavioural therapy; and (3) had taken antibiotics in the past 3 months. Group sessions and data collection took place at a clinical setting in London, UK.

#### Sample recruitment

Participants were recruited through convenience sampling via information and flyers distributed by ADHD support groups in London, online support groups and social media. The intention was to include the first 10 participants who were interested in taking part and eligible to do so. It was deemed to be important, from an ethical perspective, that this diet was trialled on a small number of participants to assess feasibility before a larger-scale RCT was conducted.

#### Sample size

The first nine participants who were interested in the study and eligible to take part (6 male; 3 female) were enrolled in the study. Participants ranged from 8 to 13 years at the commencement of the trial, with a mean age of 10.39 years (SD 1.67).

#### Dietary intervention

With guidance from a Registered Nutritional Therapist, we developed a microbiome-targeted dietary intervention specifically for this study. This is based on five action points which are described in Table 1 and were followed by participants for 6 weeks.

**Table 1:** Dietary intervention

**Action point**	**Evidence for change in microbiome**
1. **Eat at least seven servings of** ***different*** **plants each day** Vegetables, fruits, pulses, beans, herbs, spices, seeds and nuts are all counted as plants.	Vegetable fibre stimulates gut bacteria [67, 85, 86]. 30+ different plant types/week increases microbial diversity [69].
2. **Eat food within a 12-h window each day**	Time-restricted eating enhances diversity of the gut microbiome [70–72].
3. **Consume 125 ml Kefir drink each day**	Daily kefir for 4 weeks increases *Lactobacillus* in gut [73].
4. **Eat a microbiome-friendly, protein-rich breakfast from a prescribed menu** A sample breakfast menu was provided (see additional file 1). Inclusion of protein and prebiotic fibre in the form of nuts, seeds, vegetables and fruit.	Protein increases satiety [87, 88]. Prebiotic fibre promotes growth of beneficial gut bacteria [67, 85, 86].
5. **Reduce consumption of added sugar and artificial sweeteners**	Low fibre/high sugar diets associated with lower diversity of gut bacteria [89, 90]. Artificial sweeteners decrease beneficial microbes [60, 91].

Parents attended four group sessions to receive information about the diet and on-going support and to provide data about their children: a 2-h session before commencing the diet; a 1-h session at the end of weeks two and four and a 2-h session at the end of week six. The sessions were delivered by a practising registered nutritional therapist, at a medical centre in London. At sessions one and two, 24-h retrospective dietary recalls were recorded and discussed to allow the nutritional therapist to tailor advice to the needs of the participants. Participants were provided with information about the dietary principles, including general recipe ideas to apply these five principles and given food demonstrations and sample tastings. Individualised recommendations were made depending on children’s specific needs, e.g. food intolerances, reluctance to try new foods etc. Ongoing support was provided throughout the study by use of a closed WhatsApp group and via email contact.

Kefir drinks were distributed to participants every 2 weeks throughout the 6-week study. Participants were asked to consume 125 ml of kefir every day for the duration of the study, which could be taken on its own, with food or combined into a smoothie. *Nourish Kefir* supplied organic cow’s milk kefir for the study free of charge. The kefir is estimated to contain approximately 50 billion live microorganisms in a 125-ml serving. Species of micro-organisms vary due to fermentation but typically include *Leuconostoc*, *Lactococcus*, *Lactobacillus*, *Bifidobacterium*, *Saccharomyces cerevisae* and the exopolysaccharide kefiran.

One child, who was unable to tolerate dairy, was provided with a coconut kefir drink. *Life shot 100 billion* high potency coconut kefir (40 ml) was mixed with *Coconut kefir Mango & Passion* (125 ml), prepared by *Rhythm Health’s* vegan water kefir cultures, containing a mixed culture of lactic acid bacteria including *Lactobacillus acidophilus*, *Lactobacillus paracasei*, *Lactobacillus rhamnosus*, *Bifidobacterium* and *Saccharomyces cerevisiae*. The product was supplied at a reduced cost for the trial by *Rhythm Health*. This dairy-free alternative had a bacterial count of an estimated 50 billion live bacteria per day.

### Assessments

#### Primary outcome

The primary outcome measure was the completion of the study, measured as the number of participants who completed the trial.

#### Adherence to diet

Adherence to diet was measured through parental completion of a daily paper diary indicating the aspects of the diet that their child complied with. This was measured as percentage adherence to each of the five aspects of the diet over the 6-week period. The potential range was 0–100%, with a higher score reflecting a greater degree of adherence. Perceived ease and difficulty of adherence to each aspect of the diet was also assessed via an end of study online questionnaire distributed through Jisc Online Surveys (Jisc, Bristol, UK). Parents were asked to indicate how easy/difficult was to adhere to each aspect of the diet measured on a 5-point Likert scale from 1= very easy to 5=very difficult.

#### Adverse events

Adverse events were recorded according to the Common Terminology Criteria for Adverse Events [92] and European Commission guidelines [93] throughout the duration of the study. Parents were asked to report any adverse events immediately to the researchers and were additionally prompted at each session to recall any adverse events.

#### Treatment acceptability scale

Parents completed a short Treatment Acceptability Scale [94] which consisted of six questions which were responded to on a 7 point Likert scale from 1 to 7. Items include, ‘how effective do you think this treatment might be?’ Item 4 ‘How likely do you think it is that the treatment may have negative side effects?’ was reverse-scored. One question of the scale was adapted to take into account the treatment being proposed by a ‘nutritional therapist’ rather than a ‘psychologist’. Scores from all items were summed to produce a total acceptability score (with a potential range of 6–42). A higher score indicates a greater degree of perceived acceptability. The scale has been reported to have acceptable internal consistency (with a Cronbach’s alpha of .81) and very high test-retest reliability (*r*=.78) [94].

#### End of study questionnaire

In order to assess parents’ experiences with, and opinions of, the dietary intervention, parents were asked to complete a short end-of-study questionnaire. The survey consisted of nine questions exploring parents’ perceived changes in children’s behaviour, mood, sleep and gut function (e.g. ‘Have you seen any positive/negative changes in your child’s behaviour during the course of the study?’) and cost implications of the study (e.g. ‘Did taking part in the study have any cost implications for your family?). Options to choose from included ‘yes’, ‘no’, ‘not sure’ and a space for additional comments was also provided. Responses to items were considered to inform the evaluation of the study and the development of the RCT.

#### Additional outcome measures assessed Time 1 and Time 2

Additional assessments (shown in Table 2) were conducted at Time 1 and Time 2 to assess the feasibility of data collection and outcome measurements for the main RCT.

**Table 2:** Outcome measures assessed for feasibility for RCT

**Outcome**	**Instrument**	**Methodological detail**
**ADHD Symptomatology**	Conners Clinical Index (CI) from the Conners Comprehensive Behavior Rating Scales [83]. (1) Parent report (2) Teacher report (3) Self-report	25 items 5 min to complete Higher *T*-score reflects greater symptomatology
**Short-term visual working memory**	Delayed Match to Sample test - Cambridge Neuropsychological Test Automated Battery (CANTAB) [95].	15 min to complete Percent accuracy and mean reaction time for correct trials calculated.
**Sleep (objective)**	Actigraphy measure of sleep duration and quality. Children wore a ‘Motionlogger Micro watch’ (Ambulatory Monitoring, Inc., Ardsley, NY) on non-dominant wrist.	Seven consecutive days. (1) Mean activity during sleep (2) Minutes spent awake during the down period (3) Sleep latency (4) Sleep efficiency (5) Wake after sleep onset (6) Sleep fragmentation
**Sleep (subjective)**	(1) The Consensus Sleep Diary [96]. (2) Parent perceptions of sleep. Child’s Sleep Habits Questionnaire - Abbreviated (CSHQ-A) [97]. (3) Sleep Self-report (SSR) Questionnaire [98].	(1) Seven consecutive days. Used to detect and remove artefacts in the actigraphy data. (2) 22 items High score indicates more disordered sleep (3) 26 items (23 scored) High score indicates more disordered sleep
**Gastrointestinal symptoms**	The Gastrointestinal Symptom Rating Scale (GSRS) [99]— interview with child.	15 items Higher scores indicate more severe symptoms.
**Microbiome analysis**	16s rRNA stool analysis using the Omnigene Gut OM-200 kit [100, 101] by Atlas Biomed [102]. Raw data analysed using the Deblur algorithm [103].	Read counts of microbial species, genera, and families calculated. Estimation of alpha-diversity used Shannon [104] diversity metrics.

### Analytical methods

Participant characteristics and outcomes were summarised using descriptive statistics: mean (standard deviation) for continuous variables and number (percent) for categorical variables. Analysis of feasibility outcomes was based on descriptive statistics. With the small number of participants in this feasibility study, there was no formal hypothesis related to efficacy and no inferential statistics were used on the data obtained, as per best practice [82].

## Results

### Recruitment and participants

The study was advertised to 230 families through two London based child ADHD support groups. Two follow-up emails were sent and information about the study was also shared on Twitter, gaining a total of 15,289 impressions. Sixteen families expressed interest in the study. Two participants were excluded for taking medication, one was excluded for being outside the age range, one was excluded because the timing of the study did not work and a further three failed to respond to further contact. The first nine who were eligible to take part were enrolled onto the study (6 male; 3 female) between November 2018 and May 2019. Participants ranged from 8 to 13 years at the commencement of the trial, with a mean age of 10.39 years (SD 1.67). All children had a previous clinical diagnosis of ADHD according to DSM-V criteria (1). One child had to take a course of antibiotics *after* enrolment onto the study and was permitted to continue with the trial, given that the primary aim of this trial was to assess feasibility. This child was excluded from the microbiome analysis.

### Primary outcome

**Fig. 1:**
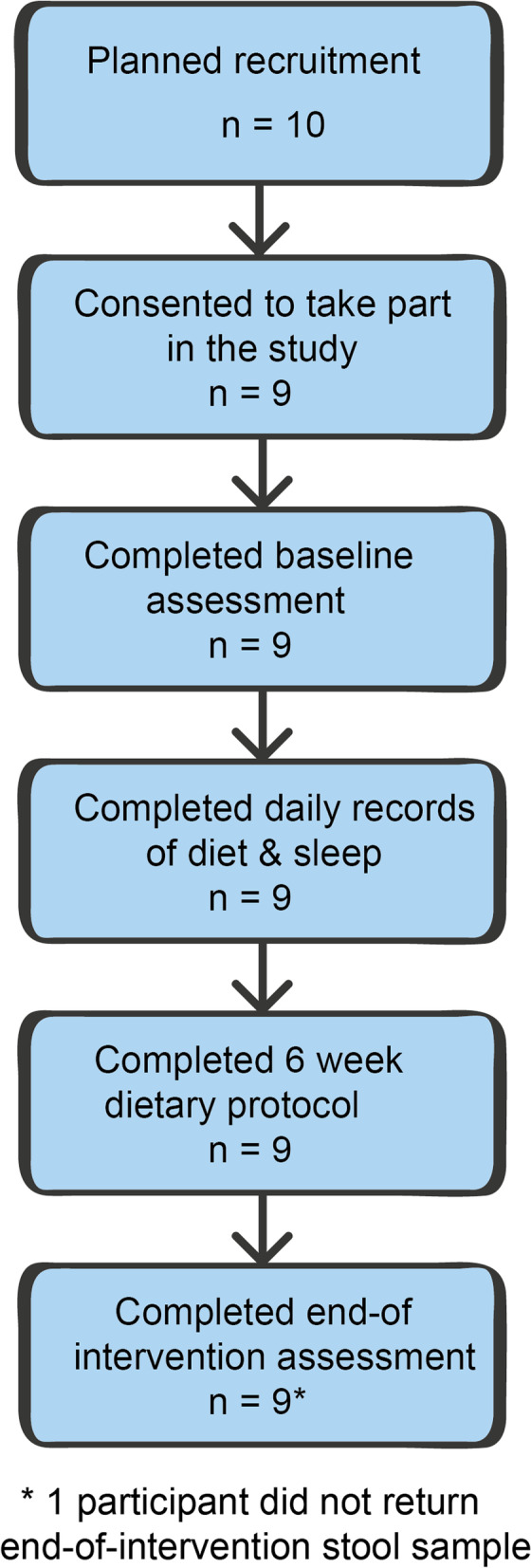
Flow of participants through the study

The primary outcome measure was completion of the study, measured as the number of participants who completed the trial. All nine participants (100%) completed the study as shown in Fig. 1. Two participants (22%) missed one task (a Conners CI teacher questionnaire and the end of study stool sample respectively). There was a small amount of Time 2 actigraphy data lost for one participant due to watch strap breakage. One stool sample was not returned at Time 2 and one additional participant was excluded from the microbiome analysis because they had to take a course of antibiotics during the intervention—the microbiome analysis was conducted on seven participants. Attendance at sessions was 100% for all except one participant, who attended two out of the four sessions and was provided with the information from these sessions through telephone consultations and emails. Data were analysed from all participants (except in the above instances). Demographic and clinical characteristics of the participants, at baseline, are shown in Table 3.

**Table 3:** Baseline demographic and clinical characteristics

**Characteristic**	**Number**	**Mean (SD)**
Age (years)		10.39 (1.67)
Sex (female)	3 (33.3%)	
Co-morbid diagnosis of Autistic Spectrum Disorder	1 (11%)	

It was difficult to recruit participants for the study. Although we cannot know for certain why the uptake for the study was low, parents who took part in the trial indicated informally that other families they knew with a child with ADHD thought a major dietary intervention would be too difficult for the family to undertake and something they did not have the skills or time to implement or that their child was a very fussy eater which would make participation difficult.

### Adherence to each aspect of the dietary protocol

**Fig. 2:**
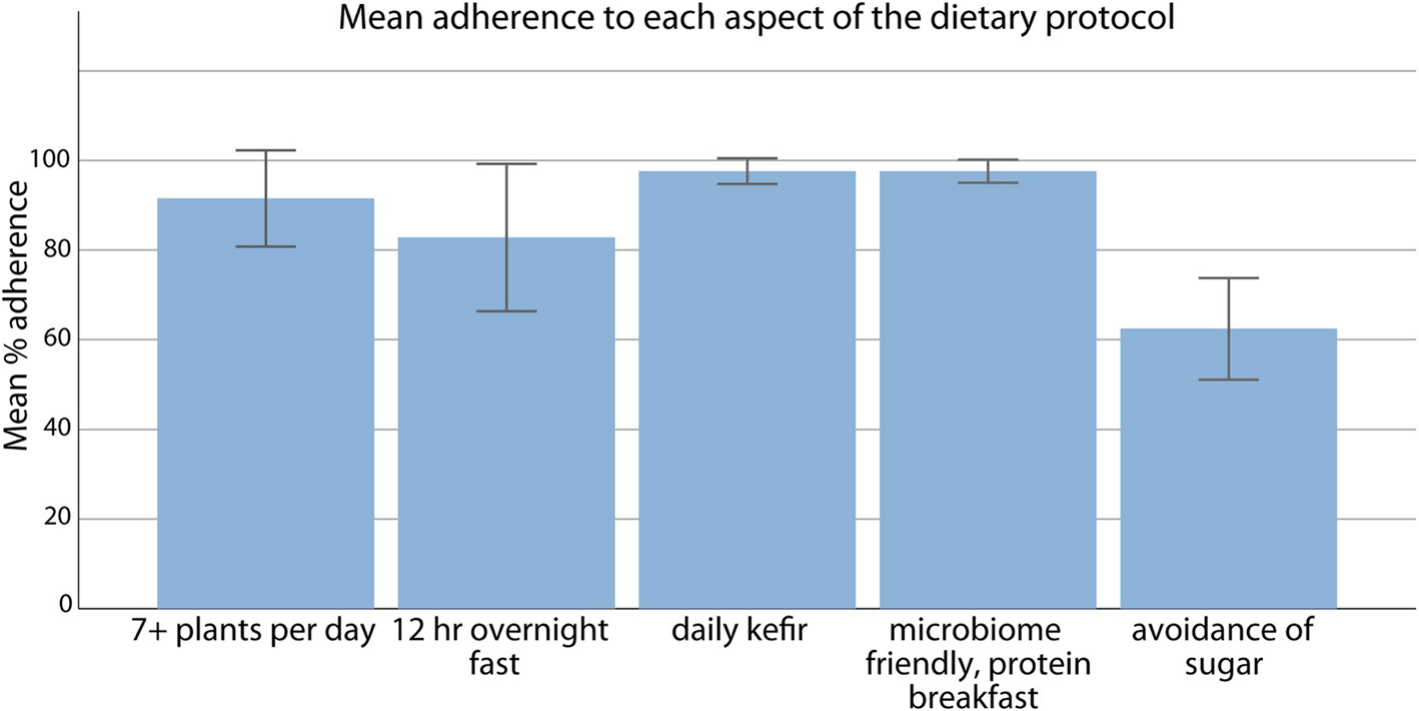
Participants’ adherence to the protocol. Error bars represent 95% confidence intervals. Percentage adherence refers to the proportion of days they complied—i.e. 41 out of 42 days (97.62%)

All participants provided completed food diaries at the relevant time points in the study. Parental report of dietary adherence to each aspect of the dietary protocol was analysed. A score of 0–100% was calculated for each participant to reflect their compliance with each of the five aspects of the diet, which was reported for each of the 42 days during the 6-week trial. Mean adherence, across all participants, is shown in Fig. 2. Perceived ease and difficulty of adherence to dietary requirements is shown in Table 4 and is broadly consistent with the actual adherence rates.

**Table 4:** Perceived ease of adherence to each aspect of the dietary protocol

Ease of adherence	Adherence to 7+ portions of plants	Adherence to 12 h overnight fast	Adherence to daily kefir consumption	Adherence to microbiome friendly, protein-rich breakfast	Adherence to avoiding sugar and artificial sweeteners
Very easy	6 (66.67%)	5 (55.56%)	6 (66.67%)	5 (55.56%)	0
Somewhat easy	1 (11.11%)	0	2 (22.22%)	3 (33.33%)	4 (44.44%)
Neither easy or difficult	0	1 (11.11%)	0	0	1 (11.11%)
Somewhat difficult	2 (22.22%)	3 (33.33%)	1 (11.11%)	1 (11.11%)	4 (44.44%)
Very difficult	0	0	0	0	0

### Adverse events

Two participants reported a mild (grade 1) adverse event during the intervention. One participant experienced a single episode of vomiting in week two of the intervention and another participant experienced a single episode of vomiting in week five of the intervention. The cause for both of these events was unknown. It was not perceived by the parents as related to the dietary changes in the study. One event was on the same day as a vaccination and the other during a period of suspected heat-stroke. Both participants continued with the intervention with no further adverse events. No other adverse events were reported during the course of the intervention.

### Parent-rated acceptability of the treatment

Mean treatment acceptability score for the adapted Treatment Acceptability Scale was 38.22 (SD = 3.49, minimum = 30, maximum = 42). The range for this questionnaire is 6–42, with a higher score indicating greater acceptability. Results indicated that parents rated the treatment closer to the ‘very acceptable’ endpoint of the dimension, than to the ‘very unacceptable’ endpoint, indicating a very good level of acceptability of the treatment.

### Parents’ perceptions of changes during the intervention

Parents’ perceived positive changes in children’s behaviour, mood and gut function are shown in Table 5. In terms of sleep, two parents reported improvements in sleep and three parents reported that children had good sleep habits prior to starting the intervention. Some parents also noted negative changes in behaviour, mood and sleep—with some noting positive and negative changes within the same category. Parents reported a positive impact of the intervention on gut function (i.e. more frequent bowel movements, less constipation, less wind and improved stool consistency) with only one parent reporting a potentially negative outcome (i.e. flatulence). The majority of the parents perceived that the study had cost implications.

**Table 5:** Perceived positive (+) and negative (−) changes in behaviour, mood, sleep, gut function and cost implications following the dietary intervention^a^

	Behaviour		Mood		Sleep		Gut function		Cost implications ^b^
	(+)	(−)	(+)	(−)	(+)	(−)	(+)	(−)	
Yes	5 (55.56%)	4 (44.44%)	5 (55.56%)	3 (33.33%)	2 (22.22%)	2 (22.22%)	5 (55.56%)	1 (11.11%)	8 (88.89%)
No	1 (11.11%)	5 (55.56%)	1 (11.11%)	6 (66.67%)	3 (33.33%)	7 (77.78%)	1 (11.11%)	8 (88.89%)	1 (11.11%)
Not sure	3 (33.33%)	0	3 (33.33%)	0	4 (44.44%)	0	3 (33.33%)	0	0

^a^Parents were asked to respond about both positive and negative changes in behaviour, mood, sleep, and gut function, eliciting 18 responses for each of these areas

^b^For cost implications, parents were just asked one question and requested to expand on their answer in a free text box. Eight parents gave a written response and all indicated that they spent more on food (during the intervention) than they would usually

### Descriptive statistics for outcome variables

The study is reported in adherence to the CONSORT extension to pilot and feasibility studies [80, 81], and as such, it is inappropriate to conduct inferential statistics on the outcome measures [82]. Descriptive data were explored for changes in outcome measures for preliminary evidence of potential effects and to guide the selection of primary outcomes for the RCT. Table 6 shows the values for the outcome measures at baseline (Time 1) and at the study endpoint (Time 2 ). The purpose of this study was not to explore efficacy. However, some of the outcome variables showed a tendency for a change in the direction of improvement in outcomes, including parent and teacher-reported ADHD symptoms and all actigraphy measures of sleep quality. There was no tendency for improvement on the computerised test of cognitive function, questionnaire measures of sleep or gastrointestinal symptoms.

**Table 6:** Baseline (Time 1) and endpoint (Time 2) descriptive statistics for outcome variables

Variable	Time 1 Mean (SD)	Time 2 Mean (SD)	Score difference (Time 2–Time 1) Mean (SD)	95% CI for mean score difference
ADHD *T*-score – Parent report	81.11 (9.17)	77.78 (10.11)	− 3.33 (7.33) ^a^	− 8.97–2.30
ADHD *T*-score – Self report	67.89 (13.11)	67.00 (15.87)	−.89 (8.10) ^a^	− 7.12–5.34
ADHD *T*-score – Teacher report	74.50 (12.95)	70.75 (14.26)	− 3.75 (11.56) ^a^	− 13.41–5.91
Delayed Match to Sample accuracy	81.11 (14.53)	77.22 (14.60)	− 3.89 (20.73) ^b^	− 19.83–12.05
Delayed match to sample reaction time (ms)	4434.63 (1346.43)	4452.36 (1483.63)	17.73 (1114.71) ^a^	− 839.11–874.57
Actigraphy – Sleep duration	601.13 (44.27)	577.57 (53.13)	− 23.56 (52.07) ^c^	− 63.58–16.47
Actigraphy – Mean activity during sleep	19.52 (5.66)	18.72 (6.40)	−.80 (3.32) ^a^	− 3.35–1.75
Actigraphy – Min spent awake during down period	116.34 (56.78)	100.62 (59.74)	− 15.72 (26.82) ^a^	− 36.34–4.90
Actigraphy – Sleep efficiency (%)	84.92 (7.89)	86.40 (8.65)	1.48 (3.76) ^b^	− 1.41–4.37
Actigraphy – Sleep onset latency	14.04 (5.83)	13.38 (6.74)	−.66 (4.13) ^a^	− 3.83–2.52
Actigraphy – Min awake after sleep onset	87.17 (49.60)	78.44 (55.28)	− 8.73 (21.36) ^a^	− 25.15–7.70
Actigraphy – Sleep fragmentation index	6.01 (2.24)	5.83 (1.98)	−.17 (1.79) ^a^	− 1.55–1.20
Actigraphy – Daytime activity	242.44 (23.45)	251.93 (18.28)	9.49 (12.98) ^c^	−.49–19.46
Child Sleep Habits (CSHQ-A) (parent-report)	44.11 (7.83)	44.44 (10.81)	.33 (7.70) ^a^	− 5.58–6.25
Sleep self-report (SSR)	36.67 (6.67)	37.56 (6.21)	.89 (3.76) ^a^	− 2.00–3.78
Gastrointestinal symptoms (GSRS)	1.44 (.51)	1.50 (.58)	.06 (.50) ^a^	−.33–.44
Alpha diversity (H)	5.64 (0.63)	5.77 (0.48)	.13 ^c^	− 0.73–0.98

^a^For these variables, a decrease reflects an improvement in symptoms

^b^For these variables, an increase reflects an improvement in symptoms

^c^For these variables, there was no set expectation with regard to direction for improvement

## Discussion

The aim of this study was to assess the feasibility of a novel microbiome-targeted dietary intervention in order to support the development of a future RCT. The study encountered recruitment challenges and it was difficult to find medication-free children whose families were willing to take on the dietary changes. However, for those who took part, the dietary intervention was acceptable, feasible, and well-tolerated in children with ADHD. All participants who enrolled in the study completed the 6-week dietary intervention and assessments, with only one participant failing to return a stool sample at the end of the intervention, and one participant missing a CI teacher report at Time 2. Adherence to the different aspects of the dietary protocol was very high.

Adherence to daily kefir and the microbiome friendly protein-rich breakfast were both high at 97.6%, with 100% adherence for more than half of the participants. Over 90% adherence was achieved for the consumption of seven or more plants per day and over 80% adherence was achieved for the 12-h overnight fast. The lowest adherence was for the avoidance of added sugar and artificial sweeteners, at just over 60%. These results were echoed in the ease of adherence feedback provided by the parents. Parents suggested that for the avoidance of sugar, social situations such as parties and school events were problematic. One parent reported that this was, ‘easy during the week when [s/he] was with me and eating at home but difficult at weekends when socialising with other children, especially at parties’. Further feedback provided in the end of study questionnaire and that came out during discussions with parents was that there were sugary snacks, sweets and cakes provided as treats at school for special occasions, other children’s birthdays, end of term parties etc., which made complete exclusion very difficult. Based on this feedback, the complete exclusion of sugar for a research trial in this age group could be very difficult to adhere to and future research should take this into consideration.

There were two isolated grade 1, mild, adverse events reported during the course of the dietary intervention. These were both single episodes of vomiting (in different participants). There was no reason to think that these adverse events were precipitated by the diet, although this cannot be ruled out. Both participants continued with the diet with no further adverse events reported for them or any of the other participants. One of the most widely used treatments for children with ADHD is medication, such as Ritalin (Methylphenidate). However, tolerability of such medication has proven to be an issue, with significant dropout rates reported [105]. The lack of severe or frequent adverse events reported for this diet, together with a zero-dropout rate in this small sample, suggest it is a safe and tolerable intervention to be further explored in children with ADHD.

Parents rated the diet as being an acceptable treatment using the Hunsley Treatment Acceptability Scale [94], with a mean acceptability score of 38.22 which was towards the upper endpoint of the scale (theoretical range 6–42). This suggests a very good degree of acceptability; there are no set points of reference for this scale but a previous study using this scale has reported a score of 33.61 as reflecting high acceptability [106]. For an intervention to have a good chance of being adopted within the ADHD population it is important that it is considered to be rated highly in terms of acceptability. Previous research has suggested that Ritalin is rated lower in comparison to behavioural interventions in terms of acceptability as an intervention for ADHD [107]. Good levels of perceived acceptability for this dietary intervention should be beneficial to recruitment of participants for an RCT.

Many families felt that following the diet had both time and cost implications. They reported spending more time on food preparation and shopping than usual and spending more money on food than they would normally. The exact increase in food cost is not something which was formally assessed. A trial implementing a modified Mediterranean diet in adults with depressive disorder found that those following the diet (which was similar in nature to that advised in this study) actually spent *less* money on their weekly shop than those in the control [108]. This possible contradiction underscores the need to test economic implications formally in future work.

One aim of this feasibility study was to evaluate data collection and outcome measures to assess their practicality for use in an RCT. Parents, teachers and participants showed an exceptionally high level of compliance for all the measures. Parents commented that online questionnaires were easier to complete than paper ones and should be considered for future studies. Participants were all compliant in wearing the ‘Motionlogger Micro watch’ to take actigraphy recordings of sleep and all were returned at the end of the study. There were three breakages of watch straps during the study. This interfered with the data collection and the 7-day period had to be re-started for two children and for one child (in the post-study recording period) was cut short resulting in data loss of 48 h. On this basis, we recommend careful assessment of watch strap durability, and possibly the use of Velcro watch straps, when carrying out actigraphy research in child participants with ADHD.

The final aim of the research was to explore the change in outcome measures for preliminary evidence of potential effects. Parent and Teacher rated mean ADHD T-scores fell within the very elevated score range (>70) at both time points, decreasing from Time 1 to Time 2 whilst self-reported scores for this measure showed minimal change. The results are suggestive that this outcome may be worth investigating further in a larger trial. Short-term visual working memory was found to be fairly accurate at both time points and did not appear to be sensitive to improvements during the course of the study. It may be worth exploring alternative neuropsychological tests, with higher rates of test-retest reliability, for any future studies. The actigraphy measures of sleep showed a consistent pattern for small improvements in sleep quality, with a reduction in total sleep duration; by improving sleep quality it can be consolidated [109]. The parent and child sleep questionnaires showed little change over the course of the study, with scores showing a marginal increase (worsening of sleep). It has been noted previously that conclusions can differ whether sleep is measured subjectively or objectively [110] stressing the importance of using different methods to assess sleep in this cohort. On this basis, we recommend the use of both actigraphy and questionnaire measures of sleep for future RCTs.

Gastrointestinal symptoms, were low at both Time 1 and Time 2. Due to the lack of severe gut symptoms, alternative questionnaires, which capture a less extreme range of gut symptoms, may be worth exploring for use in a future RCT in this population. The initial exploratory analysis of the microbiome test results highlighted a modest change in the alpha diversity score from Time 1 to Time 2. Several studies describe low alpha diversity as a factor contributing to the pathogenesis of ADHD [47, 111, 112] with recently published clinical evidence indicating that significantly lower Shannon index alpha diversity scores are seen in young ADHD patients compared to healthy controls [48]. Noteworthy observations at the family level include changes in Lachnospiraceae, Roseburia and Blautia, Bifidobacteriaceae, Sutterella, Ruminococcaceae and Bacteroides. These are beyond the scope of this article but suggest the importance of detailed microbiome exploration in future research. In terms of methodology, 16S rRNA sequencing methodology was adequately sensitive for detecting variations in microbial diversity and composition in this initial pilot study. However, many of the microbes were identified as ‘unspecified’ members of a particular taxon. Shotgun metagenomics, which sequences all genomic DNA from a sample [113] and provides greater specificity, may be a more appropriate sequencing method for a larger scale RCT for its scope to provide a greater level of depth in unravelling a mechanistic link between diet and ADHD.

This feasibility study has a number of limitations which need to be considered when interpreting the findings. First, the trial was run on a very small number of participants who may not be representative of the wider ADHD population. Second, the families who took part in the trial all appeared to be highly engaged and motivated to adhere to the dietary intervention which was a crucial factor in achieving the good adherence rates. Dietary interventions require an investment of time, energy and will-power from both the child and caregivers and as such the family situation and motivational factors could have profound implications on the degree to which a child is able to comply with the diet. A third limitation is that parents and participants were not blinded to the condition. As this study was assessing feasibility rather than efficacy, this is not a major concern but it may mean that demand characteristics are present on the subjective measures. Interestingly, where both objective (actigraphy) and subjective (questionnaire) measures were used, it was the objective measures which showed a more consistent trend for improvement. It may be important to consider, for future trials, whether it would be more manageable to focus on one aspect of the diet that had very high levels of compliance, such as the inclusion of seven or more servings of plants, the microbiome friendly, protein-rich breakfast or the consumption of kefir. Supplementation with kefir could most easily be paired with a well-matched control condition and may be less demanding on families. This could facilitate recruitment, which was problematic for this study, and increase the chances of running an RCT in a time-efficient manner.

## Conclusion

The main aim of performing this feasibility study was to pilot several components of the trial prior to the development of an RCT. To our knowledge, this study provides the first evidence of the feasibility of implementing a microbiome-targeted dietary intervention in children with ADHD. Recruitment of participants into this trial was challenging, which is an important consideration for planning future studies. The primary aim of the current study was to assess what proportion of the participants completed the 6-week dietary intervention. A completion rate of 100% suggests that, in highly motivated families, following this dietary intervention for 6 weeks is achievable and realistic. There were a number of secondary aims: (1) Percentage adherence to each aspect of the dietary protocol was assessed and found to be very good with patterns of adherence revealing what aspects of the diet might be easier to achieve compliance for in future studies; (2) Two mild adverse events were recorded during the course of the trial (which were not likely directly related to the intervention), suggesting the diet would be safe to trial on a larger population; (3) Parent rated acceptability of the treatment was found to be very good indicating that the diet is considered to be an acceptable treatment in this small group of families; (4) Data collection and outcome measures were evaluated to assess their feasibility for use in an RCT – we propose the use of online questionnaires and actigraphy watches with Velcro straps would be beneficial to implement in future research; (5) Although no formal inferential statistics were used for efficacy testing in this small sample, many of the outcome measures showed interesting patterns of results for ADHD symptoms, sleep and gut microbiome. Overall, the results support the further exploration of a microbiome targeted dietary intervention in children with ADHD. This study provides the first evidence of the feasibility of implementing a microbiome-targeted diet, supplemented with kefir, in children with ADHD. Recruitment was challenging and it is suggested that a future larger-scale study may benefit from using a dietary intervention that requires less parental time and input, in order to recruit an adequate number of participants. 

## Supplementary Information

**Additional file 1.** Sample breakfast menu.

## Data Availability

The datasets supporting the conclusions of this article are available in the FigShare repository at 10.6084/m9.figshare.13991150 and 10.6084/m9.figshare.14980116.v1.

## Abbreviations

ADHD: Attention-deficit/hyperactivity disorder
CANTAB: Cambridge Neuropsychological Test Automated Battery
CI: Clinical Index
CSHQ-A: Child’s Sleep Habits Questionnaire – Abbreviated
GSRS: Gastrointestinal Symptom Rating Scale
PUFAs: Polyunsaturated fatty acids
RCT: Randomised controlled trial
SSR: Sleep self-report
