# Supplementary material for: Trialling a microbiome-targeted dietary intervention in children with ADHD—the rationale and a non-randomised feasibility study
Source: Pilot Feasibility Stud. 2022 May 23;8:108. doi: 10.1186/s40814-022-01058-4 (PMC9125862; doi:10.1186/s40814-022-01058-4)
Supplement: Supplementary file 1 — Additional file 1. Sample breakfast menu. [file 40814_2022_1058_MOESM1_ESM.docx]

All parents were asked to include ingredients from each column of the menu. The child could opt for either a smoothie or cooked breakfast, or both, depending on their appetite but only one portion of kefir.

Sample breakfast menu

| **Menu options** | **Protein and prebiotics** | **Plants** | **Probiotics^c^** |
| --- | --- | --- | --- |
| Smoothie menu^a^ | 1 tablespoon ground flax seeds | A handful of mango (fresh or frozen) | 125ml kefir |
|  | 1 tablespoon of almond butter | Half a banana | 125ml kefir |
|  | 1 tablespoon pumpkin seeds | 2-3 chunks of fresh pineapple | 125ml kefir |
|  | 1 tablespoon of walnuts | A handful of berries (fresh or frozen) | 125ml kefir |
| Cooked Breakfast menu^b^ | 1 scrambled egg on toast (with real butter) | An apple | 125ml kefir |
|  | 1 slice of smoked salmon on toast (with real butter | A banana | 125ml kefir |
|  | 50g of tinned fish (sardines, mackerel, salmon) with hash brown | A handful of any fruit | 125ml kefir |

^a^Choose one option (or an equivalent) from each column and mix in blender or eat from bowl

^b^Choose one option (or an equivalent) from each column

^c^ Just one 125ml serving of kefir per day
